# Long-term in situ ruminal degradation of biodegradable polymers in Holstein dairy cattle

**DOI:** 10.3168/jdsc.2022-0319

**Published:** 2022-12-22

**Authors:** Hailey Galyon, Samuel Vibostok, Jane Duncan, Gonzalo Ferreira, Abby Whittington, Rebecca Cockrum

**Affiliations:** 1 School of Animal Sciences, Virginia Tech, Blacksburg 24061; 2 Department of Macromolecular Science and Engineering, Virginia Tech, Blacksburg 24061; 3 Departments of Chemical Engineering and Materials Science and Engineering, Virginia Tech, Blacksburg 24061

## Abstract

Using biodegradable materials such as polyhydroxyalkanoates (PHA) and poly(butylene succinate-*co*-adipate) (PBSA) to develop single-use agricultural plastics like bale netting may reduce the negative effects of plastic accumulation in the rumens of cattle. The objective of this research was to assess the long-term degradation of PHA, PBSA, and a PBSA:PHA blend (Blend) compared with a low-density polyethylene (LDPE) control. Polyhydroxyalkanoate, PBSA, Blend, and LDPE films were incubated in the rumens of 3 cannulated, nonlactating Holsteins for up to 150 d. In situ disappearance (ISD) and residue length were assessed after every incubation time. Data were analyzed with PROC MIXED in SAS and adjusted by Tukey's method to determine least squares differences between polymer treatments, incubation time, and their interaction. By 30 d, PHA achieved 100% degradation, with initiation occurring at 14 d indicated by ISD and a reduction in residue length. Poly(butylene succinate-*co*-adipate) and Blend did not achieve any significant ISD, but fragmentation of PBSA occurred at 60 d and fragmentation of Blend at just 1 d, likely due to abiotic hydrolysis. Low-density polyethylene achieved no ISD, and residue length did not change over incubation time. We propose that a PBSA:PHA blend is a valid alternative to polyethylene single-use agricultural plastic products based on its fragmentation within 1 d of incubation.

## Highlights

• Traditional plastic did not degrade in the rumen after 150 d of incubation.
• Biodegradable polymers demonstrated significant ruminal degradation within 60 d.
• A biodegradable polymer blend fragmented by 29% within 1 d in the rumen.
• Biodegradable polymers can potentially reduce the incidence of plastic impaction.

## Graphical Abstract

**Figure:**
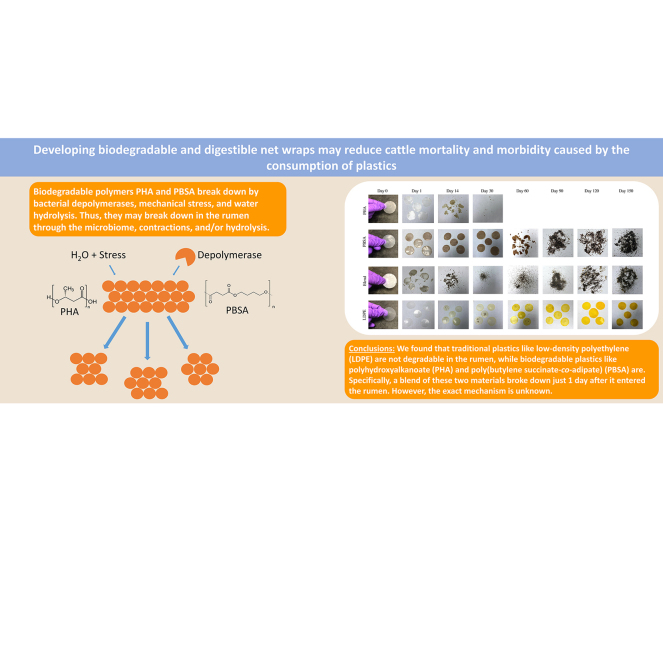
**Summary:** Developing biodegradable and digestible net wraps may reduce cattle mortality and morbidity caused by the involuntary consumption of agricultural plastics. Biodegradable materials of interest include polyhydroxyalkanoates (PHA) and poly(butylene succinate-co-adipate) (PBSA). Bacterial depolymerases, aided by mechanical stress and hydrolysis, degrade these products. Thus, these materials may break down in the rumen when consumed, unlike traditional polymers such as low-density polyethylene (LDPE). In this study, 3 cannulated Holstein dairy cows had samples of PHA, PBSA, a blend of PHA and PBSA (Blend), and LDPE incubated in the rumen for 150 days. It was demonstrated that LDPE does not break down in the rumen while biodegradable alternatives PHA and PBSA do over 150 days, with a blend composed of the 2 breaking down within just 24 hours.

Approximately 20% of domestic cattle in abattoirs have foreign bodies within the rumen, of which 50 to 60% are plastic-based materials (Mushonga et al., 2015). Involuntary plastic consumption can lead to decreased ruminal movement and microbial activity (Akraiem and Abd Al-Galil, 2016), erosion and ulcerations of ruminal papillae (Otsyina et al., 2017), and increased heavy metal concentrations within the rumen fluid (Mahadappa et al., 2020). One approach to reduce the incidence of plastic accumulation in cattle forestomachs is to develop net wrap from an alternative plastic that is biodegradable and digestible. Materials of interest include polyhydroxyalkanoate (**PHA**) and poly(butylene succinate-*co*-adipate) (**PBSA**).

Polyhydroxyalkanoates are biodegradable polymers produced within various microorganisms (Shen et al., 2009; Mudenur et al., 2019). Due to their biodegradability in various environments, chemical diversity, insolubility in water, biocompatibility, and lack of toxins, PHA polymers have a variety of applications, including food-safe storage, agricultural mulch films, and medical supplies (Roohi and Kuddus, 2018; Mudenur et al., 2019). However, PHAs are expensive and tend to be mechanically brittle. Blending PHAs with other biodegradable polymers like PBSA improves their mechanical properties, biodegradability, and production costs (Shen et al., 2009).

We hypothesize that the rumen environment may foster the degradation of PHA and PBSA through a combination of abiotic and biotic factors. Mechanical stress and increased temperatures initiate the biodegradation process by inducing fracturing of the polymers, but these factors cause minimal mass loss (Lucas et al., 2008; Salomez et al., 2019). Mass loss of polymers is largely due to microbial production of extracellular depolymerases that cleave polymer chains (Liu et al., 2010; Roohi and Kuddus, 2018). Many depolymerases that degrade PHAs and PBSA are produced by *Proteobacteria* (Nakajima-Kambe et al., 2009, Shah et al., 2013, Vigneswari et al., 2015). As *Proteobacteria* is one of the more abundant phyla present within the rumen (Van Soest, 1994), ruminal microbes may have the ability to degrade and induce mass loss of biodegradable polymers.

We previously evaluated in vitro digestion kinetics of PHA, PBSA, and a PBSA:PHA blend after fermentation in rumen fluid over 240 h (Galyon et al., 2022). The results of that experiment indicated that the beginning stages of degradation may occur within 24 h of fermentation in rumen fluid. However, mass loss was less than 1% for all polymers by 240 h and the fastest disappearance rate was 0.0031%/h. Those results could have been undermined by the limitations of an in vitro system.

The objective of this research was to assess the long-term digestibility of a commercial PHA product, PBSA, a blend of these, and a polyethylene control via in situ fermentation in ruminally cannulated Holstein cows over 5 mo. We hypothesized that PHA, PBSA, and the PBSA:PHA blend would degrade in the rumen, whereas polyethylene would not.

The Institutional Animal Care and Use Committee of Virginia Tech approved all procedures involving dairy cows (IACUC #20-208).

Proprietary PHA-based polymer (Mirel P1004) nurdles produced by Metabolix Inc. were purchased from Alterra Plastics. Poly(butylene succinate-*co*-adipate) (BioPBS) nurdles were purchased from Mitsubishi Chemical Performance Polymers. To develop the PBSA:PHA polymer blend (90% wt/wt PBSA, 10% wt/wt PHA), PHA (Mirel P1004) and PBSA (Bionolle 3001MD) were melt-blended and extruded into a nurdle formation using a pilot-scale extruder at Alterra Plastics. Low-density polyethylene (**LDPE**) was purchased from Sigma-Aldrich Inc.

Films were produced from polymer nurdles using a melt press (#SP210C-X351220, PHI Hydraulics Inc.). Approximately 100 mg of nurdles was placed between 2 nonstick Kapton sheets on platens set to 174°C and incremental pressure to 340 kg. The polymer nurdles were melted for approximately 30 s to form films 0.1 mm thick and 30 to 45 mm in diameter.

For each of the polymer samples, 24 porous Dacron bags (R510, Ankom Technology) were filled with 5 polymer films (approximately 0.50 g) and heat sealed. Bag dimensions were 5 cm wide and 10 cm long, and bag pore size was 50 ± 10 μm. In addition to 3 empty bags that served as blank samples, 3 replicates of each of the 4 polymer samples were placed in each of eight 30.5-cm × 38-cm mesh laundry-type bags. At 1300 h on the test day, all bags were simultaneously immersed within the rumens of 3 cannulated and nonlactating Holstein cows on pasture such that all cows received 8 mesh bags, each containing all treatments in triplicate. Bags were incubated for 0, 1, 14, 30, 60, 90, 120, and 150 d. For the 0-d incubation, bags were immersed into the liquid phase of the ventro-distal cavity of the rumen and extracted after 30 s. Once the incubations were finished, bags were removed and rinsed ≥3 times (3-min washing + spinning cycles) using a washing machine (SKY2767, Best Choice Products) until the rinse water ran clear. Residues were then dried in a forced-air oven at 55°C for 24 h. Residues were weighed and corrected for the mass changes of respective blank bags during fermentation within incubation time and cow. In situ disappearance (**ISD**) was calculated according to Equation [1]:

[1] $$\begin{array}{c} ISD\;\left(\%\right)=\;\\ \frac{Initial\;DM\;\left(g\right)\;-\;Corrected\;undigested\;residue\;\left(g\right)}{Initial\;DM\left(g\right)}\times 100. \end{array}$$

Based on ISD of polymers, digestion kinetic parameters for PHA were estimated using the NLIN procedure in SAS 9.4 (SAS Institute Inc.) according to the predicted digestibility Equation [2] (Ørskov and McDonald, 1979):

[2] ISD (%) = *A* + *B* × [1 – *e*^(−^*^k^*^×^*^T^*^)^],

where *T* is the time of incubation (d), *A* is the pool of immediately degraded material (%) at *T* = 0, *B* is the pool of potentially available material between 0 and 150 d (%), and *k* is the fractional rate of disappearance (%/d) of pool *B*. Pool *B* was estimated to be (100 – *C* – *A*), where *C* is the pool of nondigested material (%). For this analysis, it was assumed that both *A* and *C* were equivalent to 0%, and *B* was equivalent to 100%.

After ISD was determined, 3 individual residue pieces were removed from each nylon bag and their length was measured. Residue length was measured using a pair of digital calipers, and the average length of polymer residues was determined per bag.

All statistical analyses were conducted with PROC MIXED, and least squares differences were adjusted by Tukey's method in SAS. Polymer ISD and residue length were both tested with the fixed effects of treatment (df = 3), time (df = 7), their interaction (df = 21), and the random residual error (df = 254), as well as the random effect of cow (df = 2). Data were evaluated with an α value of 0.05.

In situ disappearance of polymers is demonstrated in Figure 1(A). There was no ISD for any polymer after only 1 d of incubation in the rumen. Neither PBSA, Blend, nor LDPE films achieved any significant ISD compared with 0 d over the entire incubation period. Films made of PHA demonstrated exponential degradation. At 14 d, the ISD of PHA films increased by 55% compared with d 0 (*P* < 0.001). From 14 to 30 d, there was an 80% increase in mass loss (*P* < 0.001) and PHA degraded by 100%. As ISD did not change across incubation time for the remaining polymers, their fractional rates of degradation were not determined. The fractional rate of degradation of PHA was 7.84%/d.

**Figure 1:**
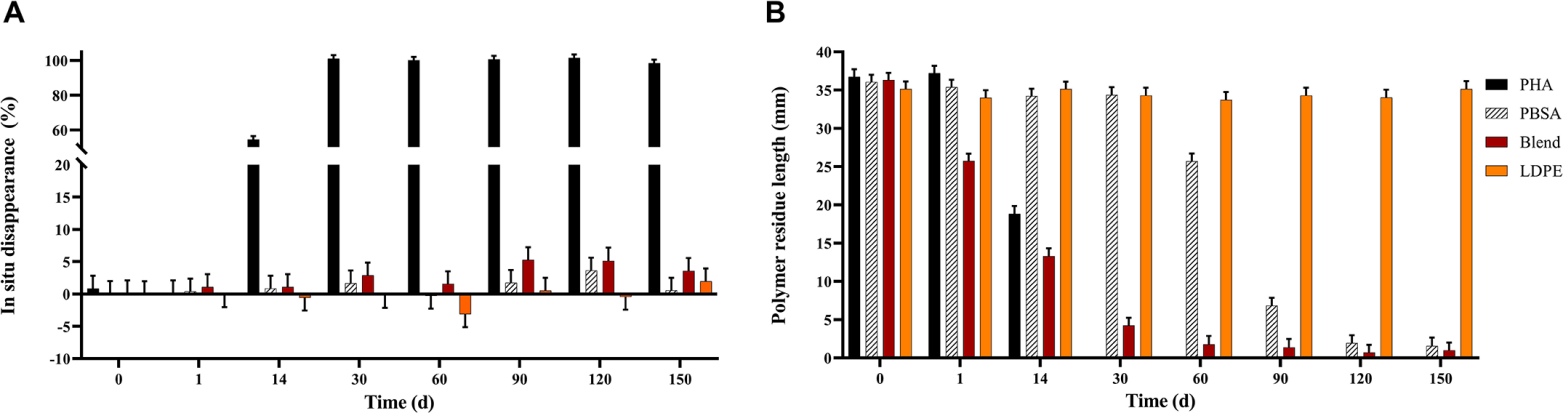
(A) In situ disappearance (%) of polyhydroxyalkanoate (PHA), poly(butylene succinate-co-adipate) (PBSA), PBSA:PHA (90% PBSA, 10% PHA, wt/wt; Blend), and low-density polyethylene (LDPE) films over 150 d. Treatment: *P* < 0.001. Time: *P* < 0.001. Treatment × Time: *P* < 0.001. (B) Residue length (mm) of PHA, PBSA, Blend, and LDPE films incubated in the rumen over 150 d. Treatment: *P* < 0.0001. Time: *P* < 0.0001. Treatment × Time: *P* < 0.0001. All data are shown as LSM ± SEM.

Upon visual assessment of polymers (Figure 2), PHA, PBSA, and Blend films became brittle and film residue size declined over incubation time. Films made of LDPE did not appear to demonstrate degradation and became stained after longer incubation times in the rumen. Polymer residue length is demonstrated in Figure 1(B). Polyhydroxyalkanoate film residue size decreased by 49% by 14 d compared with 0 d (*P* < 0.0001). Due to complete degradation of PHA films by 30 d, the length of PHA residues from 30 d onward is unavailable. Residue length of PBSA decreased by 29% by 60 d compared with 0 d (*P* < 0.0001). The PBSA films then decreased by an additional 7% to 6.84 mm by 90 d compared with 60 d (*P* < 0.0001). The film residue length of PBSA did not decrease thereafter, although there was a tendency to decrease another 7% by 150 d compared with 90 d (*P* = 0.08). Blend film residue length decreased by 29% by 1 d compared with 0 d (*P* < 0.0001). Blend films continued to fragment into smaller particles consecutively. Residue length decreased down to 13.30 mm by 14 d (*P* < 0.0001), and then to 4.23 mm by 30 d (*P* < 0.0001). Although residue length was reduced to 0.65 mm by 150 d, length did not significantly change after 30 d. The LDPE films did not decrease in residue length throughout the study.

**Figure 2:**
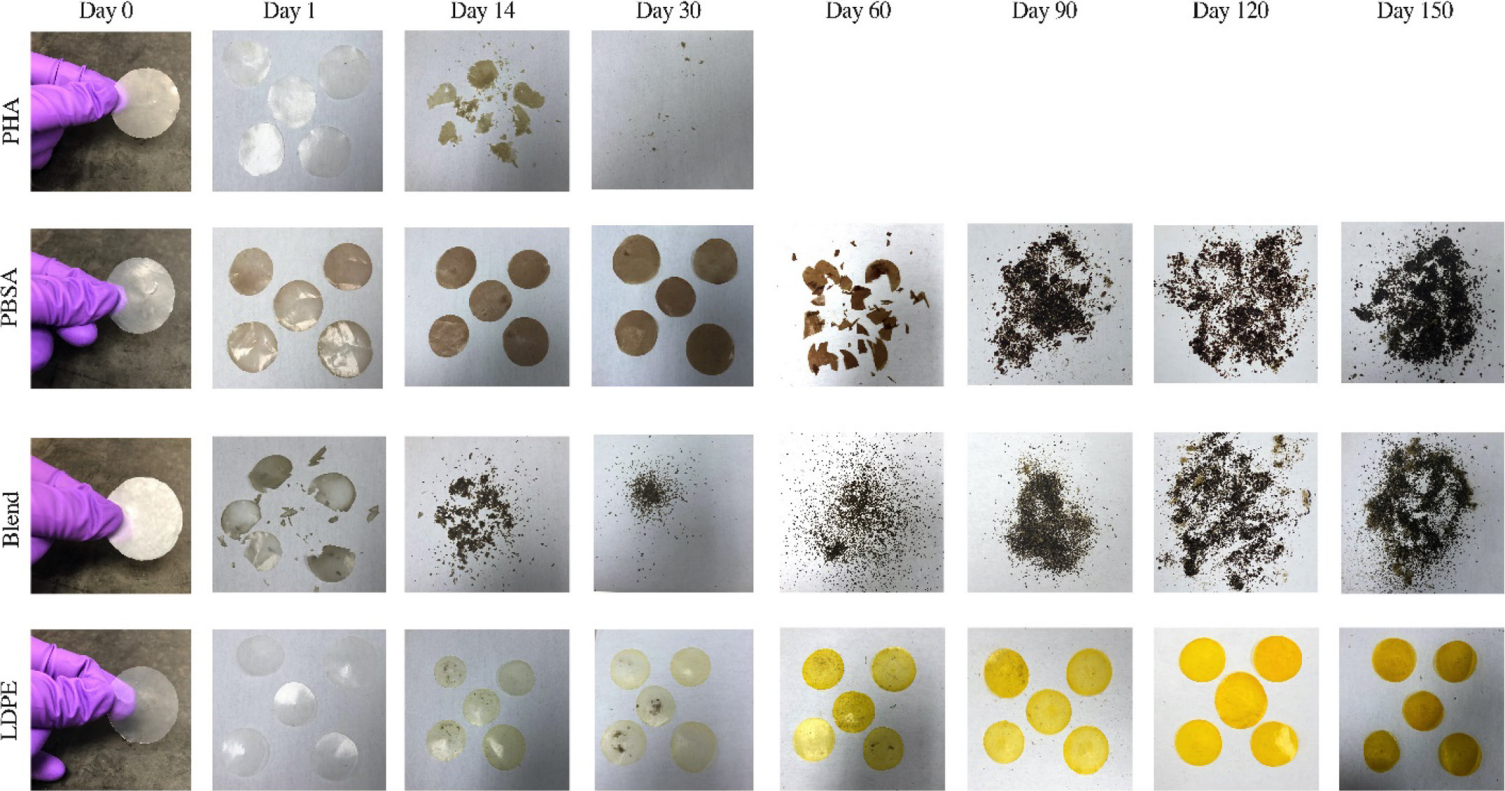
Visual assessment of polyhydroxyalkanoate (PHA), poly(butylene succinate-co-adipate) (PBSA), PBSA:PHA (90% PBSA, 10% PHA, wt/wt; Blend), and low-density polyethylene (LDPE) films incubated in the rumen over 150 d.

Our findings demonstrated that the ruminal environment is highly capable of degrading PHA- and PBSA-based biodegradable polymers. Compared with the results of our in vitro fermentation study of these products, in situ degradation was increased. In vitro degradation of PHA resulted in 0.53% mass loss by 10 d, with a fractional disappearance rate of 0.002%/h (Galyon et al., 2022), whereas in situ degradation of PHA resulted in a remarkable 55% mass loss by 14 d and a fractional degradation rate of 7.84%/d.

Possible explanations for increased degradation in an in situ model include continuous inoculum and increased motility. The Daisy^II^ Incubator in vitro method is a batch method, and the microbial population changes over time without new inoculum. Within just 48 h of in vitro culture in the Daisy^II^ Incubator, the total rumen fluid bacteria population declines and leaves only 0.5 of the original population (Soto et al., 2013). It is plausible that bacteria in rumen fluid with the potential to degrade these biodegradable polymers are not fully retained in the batch system to sufficiently degrade and induce mass loss. An in situ model allows for continuous inoculation and introduction of new feedstuffs to support proliferation. This environment likely supports the degradation of PHA and PBSA materials if they rely on bacterial hydrolysis by sustaining bacterial populations. Further, mechanical stress placed on incubated polymers is increased in an in situ model due to ruminal contractions. This could lead to abiotic fragmentation and increased surface area for improved bacterial hydrolysis.

Additionally, polymer formation utilized in this study may have contributed to increased mass loss. Polymers in this study were pressed into films that were 0.1 mm thick and approximately 35 mm in diameter, whereas our in vitro study evaluated polymers as granules that were approximately 3 mm in diameter. A study of poly(3-hydroxybutyrate) and a copolymer with poly(3-hydroxyvalerate) in tropical coastal waters over 160 d found that films that were 0.1 mm thick with a 30-mm diameter had 53 and 315% increases in mass loss, respectively, compared with 10-mm pellets of the same chemical structures (Volova et al., 2010). Increased mass-to-surface area ratio likely allows for a greater interface between the polymer surface and the microbial consortium within the rumen. This would enhance bacterial attachment and enzymatic hydrolysis via extracellular depolymerases. This could explain, in part, the great increase of biodegradation in this study compared with our previous in vitro model.

Previous studies indicated there may be a lag phase of biodegradation of PHA-based materials, allowing time for bacterial attachment to polymer surfaces. Depending on the microbial population and polymer composition, the lag or stationary phase can be anywhere from a few days to months (Volova et al., 2010; Wen and Lu, 2012). It is possible that at 10 d of incubation within rumen fluid, polymers are still in the stationary phase, when little to no mass loss occurs. The lag phase could be overcome between 10 and 14 d of incubation in rumen fluid, leading to the detected exponential increase in ISD. Further studies in which in situ biodegradation of commercial PHA is more readily evaluated between 1 and 14 d are needed to detect whether 2 periods of mass loss occur for a proprietary PHA blend.

Although PHA digestibility was greatly improved, neither PBSA nor PBSA:PHA mass disappearance was improved by an in situ approach for longer durations. However, we observed a significant decrease in polymer residue length of PBSA by 60 d and of the Blend (90% PBSA) by just 1 d of incubation in the rumens of dairy cattle. Comparing the trends of ISD and residue length indicates that synthetic biodegradable polymers such as PBSA and blends mostly comprising synthetic biodegradable polymers may degrade more by abiotic factors in the rumen.

Hydrolytic cleavage of polymer chains occurs when water molecules penetrate the amorphous region and reduce molecular weight to induce fragmentation. Through this process, intact polymers burst into smaller fragments, although overall mass loss does not necessarily occur (Hakkarainen, 2002; Salomez et al., 2019). Enzymatic degradation of polymers initiates at a slower rate and hydrolyzes polymer chains at the surface level due to enzymes being too large to enter the amorphous region (Hakkarainen, 2002; Lucas et al., 2008). Oligomeric and monomeric constituents are released, and mass loss of the polymer is detected. Thus, we propose that the fragmentation of PBSA and Blend was largely induced by spontaneous water hydrolysis in rumen fluid, whereas PHA biodegradation may have occurred largely through enzymatic hydrolysis. This is supported by visual appraisal of polymer residues. The 14-d residues of PHA were porous with apparent organic degradation of the outermost edges, whereas PBSA at 60 d and Blend at 1 d had brittle residues with sharp, geometric edges and no visible appearance of surface erosion. Further studies using scanning electron microscopy of polymer surfaces after incubation are needed to confirm whether surface erosion is present after fermentation.

Comparing PBSA alone to Blend, polymer fragmentation occurred at a much slower rate, even though the mechanism of degradation was likely the same. The improved fragmentation was likely due to the inclusion of PHA. A study in which polybutylene succinate and PBSA underwent biodegradation in compost found that polybutylene succinate took 6 wk to begin to fragment, whereas the copolymer with adipate (PBSA) began to fragment at just 4 wk (Puchalski et al., 2018). The trend that copolymers degrade more readily than their constituents holds true throughout the literature (Mergaert et al., 1993; Sridewi et al., 2006; Kupczak et al., 2021).

In terms of animal ingestion, a PBSA:PHA blend is a promising alternative to the typical polyethylene often used for bale netting. Although ISD was not significant, polymer residue size began decreasing within 24 h of incubation. Polyethylene products remain in the rumen and negatively affect animal health because they cannot break down sufficiently to pass through to the omasum (Pizol et al., 2017). Based on our findings, we suggest that bale netting developed from a PBSA:PHA blend will be less harmful than polyethylene if consumed due to the fragmentation of the material. Particles <1.18 mm are more readily able to escape the ruminoreticulum to the omasum (Poppi et al., 1981). Thus, we conclude that a PBSA:PHA blend should be able to escape the ruminoreticulum by 150 d, if not before. Further studies are needed to determine the exact mechanisms underlying PHA and PBSA degradation in the rumen environment. Future feeding studies are necessary to elucidate the ability of biodegradable polymers to pass through the digestive tract and the subsequent health of animals with long-term consumption.
